# Scar Formation with Decreased Cardiac Function Following Ischemia/Reperfusion Injury in 1 Month Old Swine

**DOI:** 10.3390/jcdd7010001

**Published:** 2019-12-18

**Authors:** Emma J Agnew, Nivedhitha Velayutham, Gabriela Matos Ortiz, Christina M Alfieri, Luis Hortells, Victoria Moore, Kyle W Riggs, R. Scott Baker, Aaron M Gibson, Sithara Raju Ponny, Tarek Alsaied, Farhan Zafar, Katherine E Yutzey

**Affiliations:** 1 Molecular Cardiovascular Biology, Cincinnati Children’s Hospital Medical Center, 3333 Burnet Ave, Cincinnati, OH 45229, USA; emma.agnew@cchmc.org (E.J.A.); nivedhitha.velayutham@cchmc.org (N.V.); gabriela.matos3@upr.edu (G.M.O.); christina.alfieri@cchmc.org (C.M.A.); Luis.HortellsGarcia@cchmc.org (L.H.); 2 Cincinnati Children’s Hospital Heart Institute, Department of Pediatrics, University of Cincinnati College of Medicine Cincinnati, Cincinnati, OH 45229, USA; Vicky.Moore@cchmc.org (V.M.); Tarek.Alsaied2@cchmc.org (T.A.); 3 Division of Pediatric Cardiothoracic Surgery, The Heart Institute, Cincinnati Children’s Hospital Medical Center, University of Cincinnati, Cincinnati, OH 45229, USA; Kyle.Riggs@cchmc.org (K.W.R.); sbaker117@gmail.com (R.S.B.); aaron.gibson@cchmc.org (A.M.G.); Farhan.Zafar@cchmc.org (F.Z.); 4 Division of Human Genetics, Cincinnati Children’s Hospital Medical Center, 3333 Burnet Avenue, Cincinnati, OH 45229, USA; SitharaRaju.Ponny@cchmc.org

**Keywords:** ischemia/reperfusion injury, cardiac function, cell cycling, cardiomyocyte, swine

## Abstract

Studies in mice show a brief neonatal period of cardiac regeneration with minimal scar formation, but less is known about reparative mechanisms in large mammals. A transient cardiac injury approach (ischemia/reperfusion, IR) was used in weaned postnatal day (P)30 pigs to assess regenerative repair in young large mammals at a stage when cardiomyocyte (CM) mitotic activity is still detected. Female and male P30 pigs were subjected to cardiac ischemia (1 h) by occlusion of the left anterior descending artery followed by reperfusion, or to a sham operation. Following IR, myocardial damage occurred, with cardiac ejection fraction significantly decreased 2 h post-ischemia. No improvement or worsening of cardiac function to the 4 week study end-point was observed. Histology demonstrated CM cell cycling, detectable by phospho-histone H3 staining, at 2 months of age in multinucleated CMs in both sham-operated and IR pigs. Inflammation and regional scar formation in the epicardial region proximal to injury were observed 4 weeks post-IR. Thus, pigs subjected to cardiac IR at P30 show myocardial damage with a prolonged decrease in cardiac function, formation of a regional scar, and increased inflammation, but do not regenerate myocardium even in the presence of CM mitotic activity.

## 1. Introduction

The ability of the heart to regenerate has been demonstrated in zebrafish, amphibians and neonatal mammals [1]. In mice and pigs, a brief neonatal window of cardiac regenerative capacity after injury has been linked to the proliferation of cardiomyocytes (CM) [2,3,4]. However, whether or not the human heart can regenerate, alongside the timing and mechanisms involved in loss of cardiac regenerative capacity in mammals, are unknown. Thus, there remains a need to further research cardiac development and repair following injury in the large mammalian setting. Here, we examine the ability of the young swine heart to repair following ischemia/reperfusion (IR) injury.

In mammals, the prenatal heart typically grows by hyperplasia, with mitosis followed by cytokinesis to produce mononucleated CMs [5]. It is known that mice can regenerate their hearts with pre-existing CMs being the source of regenerating cardiac muscle following injury in the first week after birth [2,6,7]. However, this intrinsic capacity for cardiac self-repair is lost by postnatal day (P)7 coincident with CM binucleation, cell cycle arrest, and the transition to hypertrophic growth [2,8,9,10]. There is incomplete information available as to how the transient regenerative capacity of the neonatal mouse heart specifically relates to CM cell cycle exit, transition to hypertrophic growth, sarcomeric maturation, and fibrotic scar formation in response to injury [2,9,11].

In large mammals, less is known of the timing of CM proliferative arrest, multinucleation, transition to hypertrophic growth, and regenerative capacity. Studies in large mammals are required for clinical translation to humans, with thorough analysis of proliferation, nucleation and ploidy across multiple postnatal stages. Recent studies from our group [12] found that the postnatal pig heart shows CM mitotic activity in the first 2 months after birth, peaking at P15, resulting in longitudinal growth of CMs by multinucleation. Sarcomeric maturation, multinucleation and hypertrophic CM growth continues to 2–6 months after birth in swine, with CM nuclei predominantly diploid from P0–6 months of age. The presence of significant numbers of mononucleated diploid CMs in pig hearts in the month after birth, alongside active cardiac cell cycling, suggests that there may be an extended period for postnatal cardiac regenerative repair via CM proliferative mechanisms after injury in swine [13].

Recent reports have demonstrated a limited regenerative period of 2–3 days after birth in neonatal pigs subjected to permanent ligation of the LAD, which leads to a severe drop in cardiac function [3,4]. Studies in adult swine show no evidence of cardiac regeneration after ischemic injury without intervention, and previous reports in pigs are primarily focused on developing therapeutic targets to aid in cardiac repair [14,15,16,17]. This study examines cardiac repair with use of an alternative, milder, injury approach to that previously assessed in young swine [3,4]. Here we report the response to transient ischemic injury at P30 when CM mitotic activity, multinucleation, and extracellular matrix (ECM) remodeling are ongoing in postnatal swine.

## 2. Materials and Methods

All animal experiments conform to NIH guidelines and were performed with protocols approved by the Institutional Animal Care and Use Committee (IACUC) at the Cincinnati Children’s Research Foundation. White Yorkshire-Landrace farm pigs were purchased from Isler Genetics (Prospect, OH, USA). All animals (n = 19 total, with n = 16 surviving the full protocol) were aged 1 month (P30) at time of surgery, following 2–3 days of acclimatization upon arrival at the Cincinnati Children’s Hospital Medical Center (CCHMC) animal facility. The weight range of pigs at time of surgery was 6–10 kg.

### 2.1. Ischemia/Reperfusion (IR) Cardiac Injury

P30 pigs were randomly assigned to sham (n = 8) or IR (n = 11) cardiac surgery groups. Left ventricular myocardial samples were also collected from unoperated 2 month old pigs (n = 4), to be used as control samples. Of the 11 pigs subjected to IR injury, 8 survived the day of the surgery and no subsequent morbidity or mortality was observed. Equal numbers of males (n = 4) and females (n = 4) were evaluated in each group. On the day of surgery, anesthesia was induced with intramuscular injection of ketamine (20 mg/kg, Zoetis, Kalamazoo, MI, USA) and xylazine (dose 2 mg/kg, Akorn Animal Health, Lake Forest, IL, USA). Each pig was intubated with an endotracheal tube of 4–5 French diameter, with anesthesia being sustained by 1%–2% isoflurane (Akorn Animal Health) with 100% oxygen. Continuous pulse oximetry measured blood oxygenation throughout the procedure. Three-lead electrocardiogram (ECG) was used to monitor for arrhythmias. A rectal probe was inserted to measure body temperature.

Prior to incision, amiodarone was given intramuscularly (1 mg/kg, APP Pharmaceuticals, Schaumburg, IL, USA). IV fluids were maintained at 30 cc/hr with 5% dextrose in Lactated Ringer’s solution. Left lateral thoracotomy was performed to approach the 4th intercostal space and the 3rd or 4th rib was removed after examining the heart’s position. After opening the pericardium, a 4-0 Prolene suture (Ethicon, NJ, USA) was placed around the left anterior descending (LAD) artery just distal to the 2nd diagonal branch. All IR pigs were administered heparin (1000 units, Sagent Pharmaceuticals, Schaumburg, IL, USA) and a rubber snare was used to occlude the LAD. ST segment changes were observed on the ECG and the myocardium was visually examined for ischemic changes distal to the spot of occlusion. LAD occlusion lasted 1 h, after which the snare was released and reperfusion was monitored for 30 min. For the sham operations, the LAD suture was not snared down. The chest was left open for 1.5 h in total, comparable to IR operations. In all pigs, an air knot was left to mark the site of injury for accurate tissue collection at euthanasia. Epinephrine (1 mg) and lidocaine (2 mL/mg)(Henery Schein, Dublin, OH, USA), cardiac defibrillation with 10J+ energy, and cardiac massage were given appropriately and as needed in all animals who developed significant arrhythmias or bradycardia during the procedure. For chest closure, the ribs were re-opposed with 2-0 Vicryl stitches and tissue closed in layers with Vicryl and Monocryl sutures (Ethicon, NJ, USA and Medline, IL, USA), followed by 1–2 mg/kg of Bupivicaine (Hospira, Lake Forest, IL, USA) was administered around the incision site.

Antibiotics were administered by intramuscular injection of Combi-Pen-48 (30,000 U/Kg, Bimeda, Oakbrook Terrance, IL, USA) on the day of and 2 days after surgery. Pain management medication was induced with intramuscular injection twice a day for two days with Buprenorphine Hydrochloride (0.01 mg/Lg, PAR Pharmaceuticals, Chestnut Ridge, NY, USA).

Two pigs in the IR group did not survive the procedure, following bradycardia at 20 and 40 min respectively post-ischemia. One IR pig showed poor recovery post-surgery and was euthanized 7 h post-ischemia. All animals that survived the day of surgery survived the full study (n = 16). One month after surgery, pigs were euthanized, via injection of Fatal-Plus (1 cc per 4.5 kg, Vortech Pharmaceuticals, Dearborn, MI, USA), for tissue collection and processing.

### 2.2. Serum Cardiac Troponin I Determination

Peripheral blood samples were collected before surgery and 2 h following IR (or sham equivalent) to measure physiological parameters, including blood chemistry and circulating cTnI, indicative of myocardial damage. Immediately following collection, blood samples were inserted into the iSTAT test cartridges and automatically read with the iSTAT blood analyzer to detect cardiac troponin I protein in serum (Abbott Laboratories Diagnostics, Lake Forest, IL, USA).

### 2.3. Echocardiography

Transthoracic echocardiography was performed prior to surgery, 2 h post-cardiac suture placement, 1 week, 2 weeks, and 4 weeks post-operatively using the General Electric Vivid 7 system (Boston, MA, USA) with Digiview software, cardiac package (Tech Tools, Rowlett, TX, USA). Briefly, pigs were anaesthetized as described above (intramuscular injection of ketamine, followed by 1%–2% isoflurane with 100% oxygen after intubation), placed in left lateral decubitus position with electrocardiogram electrodes attached to monitor heart rate and a rectal probe inserted to observe body temperature. The sonographer was blinded to the experimental groups during all imaging and data analysis. Images were acquired, with a 5 MHz probe, in 2D mode short and long axis, with M-mode images taken in short axis. Tissue doppler imaging measurements of the septal and lateral wall of the left ventricle (LV) were made. Two-dimensional parasternal long axis four chamber images were analyzed for LV diastolic and systolic dimensions (cm) and volumes (mL), stroke volume (mL), fractional shortening (%) and ejection fraction (%). M-mode images taken at the mid-papillary muscle end were utilized for analysis of left ventricular septum diastolic and systolic thickness (cm) and LV posterior wall diastolic and systolic thickness (cm). Heart rate in beats per minute (bpm) was obtained from M-mode images. Digiview analysis software was used for calculating all above parameters.

### 2.4. Histology and Tissue Staining

For tissue processing, 3 zones (approximately 1 cm cubes) of P30 pig myocardium 4 weeks post-surgery were collected. The scar zone was defined as the region directly inferior to the occlusion site where loss of blood flow and myocardial ischemic damage would be expected to occur. Upon processing, it was noted this area contained both scar tissue and adjacent myocardium in IR pigs. In sham pigs, the same size piece directly at the occlusion site was taken and named ‘scar’ for regional comparisons. The border zone was defined as the area of the LV adjacent to the area classified as ‘scar’. The remote zone was taken from the posterior side of the middle LV. Myocardium was fixed in 4% Paraformaldehyde (PFA) (Electron Microscopy Sciences, Hatfield, PA, USA) in Phosphate-buffered saline (PBS) (Fisher Scientific, Hampton, NH, USA), then transferred to 70% ethanol after 48 h. Tissues were processed, embedded in paraffin wax and cut using a microtome to 5 µm sections of the myocardium, encompassing both the endo- and epicardial regions.

#### 2.4.1. Masson’s Trichrome

Masson’s Trichrome 2000 Stain Kit (American Master Tech Scientific, McKinney, TX, USA) was used to identify collagen and muscle in all myocardial sections, according to manufacturer’s procedures. Brightfield images were obtained with an Olympus BX51 microscope and Nikon DS-Ri1 camera (both Tokyo, Japan). Deposition of collagen was quantified on an automated analysis program created on NIS elements software (Tokyo, Japan).

#### 2.4.2. Immunohistochemistry

Sections were dewaxed with xylene (Fisher Scientific, Hampton, NH, USA) and rehydrated in decreasing concentrations of ethanol (Decon Labs Inc., PA, USA). Antigen retrieval was carried out in citrate buffer (pH 6; Vector, Burlingame, CA, USA) using a pressure cooker. Slides were blocked with 6% goat serum, followed by staining with Anti-phospho-Histone H3 (Ser10) Antibody (pHH3, 1:100; 06-570, Millipore Sigma, Burlington, MA, USA) or CD45 Antibody (1:200; ab10558, Abcam, Cambridge, UK), in combination with the cardiomyocyte marker Anti-Troponin I Antibody (cTnI, 1:1000; MAB1691) (Millipore Sigma). Fluorophore-conjugated secondary antibodies (1:100; Alexa Fluor ab175473, ab150081, Abcam, Cambridge, UK) were used to detect immunostaining, with the addition of wheat germ agglutinin (WGA), Alexa Fluor™ 647 Conjugate (1:250; Thermo Fisher Scientific, Waltham, MA, USA) and DAPI (5 mg/mL, D1306; Thermo Fisher Scientific). Immunofluorescence was imaged using a Nikon Eclipse Ti Fluorescence microscope with NIS elements software (Tokyo, Japan). The percent pHH3-positive cardiomyocytes, normalized to the total number of cardiomyocyte nuclei, from cross-sectional images, was calculated using an automated analysis program created on NIS elements software. Fiji (ImageJ) image analysis software was used to manually draw the WGA-defined circumference of cardiomyocytes with a central nucleus to calculate cross sectional area (µm^2^).

#### 2.4.3. Lectin-DAB Staining

Glycoprotein lectin was used to identify capillary endothelial cells in all myocardial sections. Sections were dewaxed, rehydrated and antigen retrieval carried out as above. Slides were blocked with 0.3% H_2_O_2_, followed by 6% goat serum. Sections were stained with Biotinylated Lectin Antibody (B-1105, Vector Laboratories, Burlingame, CA, USA) in a 1:300 dilution. Following overnight incubation, mouse Avidin-Biotin Complex (ABC; Thermo Scientific) was added to the stained sections. The chromogen used for visualization of the lectin signals was 3,3′-Diaminobenzidine (DAB), made using DAB metal concentrate (10x in stable peroxide substrate buffer (1x) at a 1:100 dilution (Thermo Scientific). Stains were imaged using an Olympus BX51 microscope and Nikon DS-Ri1 camera with NIS elements software. Microvessel density, measured as small vessel counts per tissue area (µm^2^), was calculated using an automated analysis program created on NIS Analysis elements software.

#### 2.4.4. Cell Death Detection

Cell death was detected by TUNEL using the In Situ Cell Death Detection Kit, Fluorescein (Roche, Basel, Switzerland). Briefly, slides were baked for 1 h following by dewaxing and rehydration. Antigen retrieval was achieved via proteinase K addition (20 µg/mL; Carlsbad, CA, USA). Slides were blocked in 4% goat serum, then incubated overnight with the monoclonal Anti-α-Actinin (Sarcomeric) antibody (A7811, 1:100)(Millipore Sigma), before the addition of TUNEL enzyme (1:10, Roche), goat anti-mouse IgG H&L (Alexa Fluor^®^ 568) (1:400, ab175473, Abcam) and wheat germ agglutinin, Alexa Fluor™ 647 Conjugate (1:250; Thermo Fisher Scientific). Cell death was imaged as above, and quantified as the number of cardiomyocytes (identified via sarcomeric α-actinin stain) and non-cardiomyocytes normalized to total nuclear number (DAPI).

#### 2.4.5. Collagen Hybridizing Peptide Detection

Slides were dewaxed and rehydrated before addition of Streptavidin reagent (Component A) for 30 min at 37 °C, followed by Component B after washing (E21390, Thermo Fisher Scientific). Slides were blocked with 5% goat serum and collagen hybridizing peptide (CHP) solution (15 µM, BIO300, 3Helix, Salt Lake City, UT) prepared by heating to 80 °C before placing in ice for 15 s prior to addition to tissue sections for overnight incubation at 4 °C. Streptavidin, Alexa Fluor™ 568 conjugate secondary antibody (1:300, Thermo Fisher Scientific) was added and tissues imaged as above. CHP was calculated as area (µm^2^) per field.

### 2.5. RNA Analysis

Myocardial tissue samples from all three zones isolated in parallel with histology samples described above were snap frozen in liquid nitrogen and stored at −80 °C. RNA was extracted using the NucleoSpin RNA kit (740955, Macherey-Nagel, Duren, Germany). The manufacturer’s protocol was followed, with elution in 40 µL RNase-free H_2_0. RNA was treated with DNase I treatment (EN0255, Thermo Fisher Scientific, Waltham, MA, USA), according to manufacturer’s protocols. cDNA was synthesized, using the SuperScript III First-Strand Synthesis SuperMix kit (Thermo Fisher Scientific), then subjected to real-time quantitative PCR (RT-qPCR) in duplicate using the Step One Plus system (Applied Biosystems, Foster City, CA, USA) with gene-specific primer sets (Table 1) and Applied Biosystems Power SYBR Green PCR Master Mix (Thermo Fisher Scientific). Porcine primers were designed on Primer-BLAST (NCBI National Center for Biotechnology Information, Bethesda, MD, USA). Primers were tested for specificity using Sanger DNA sequencing (performed by CCHMC DNA Core) of pooled cDNA sample PCR products, following purification with use of the QIAquick PCR Purification Kit (Qiagen, Hilden, Germany). Relative quantification was provided by StepOne software v2.3 using the quantitation-relative standard curve method with 40 cycles followed by melt curve production. mRNA levels were normalized to the mean *18S* rRNA levels, used as internal standards, and expressed relative to unoperated 2 month old pig myocardial samples.

#### 2.5.1. RNA-Sequencing

RNA sequencing (RNA-seq) was performed using the Illumina HiSeq 2500 system in collaboration with the CCHMC DNA Core. Total RNA from pig myocardial tissue was subjected to the TruSeq mRNA LS Illumina protocol with a GeneAmp 9700 Applied Biosystems thermocycler. All sample/library quality control analysis was carried out on the AATI Fragment Analyzer (Agilent) and quantified using a Qubit fluorimeter (Thermo Fisher Scientific). Adapter dimers in the libraries were removed from the pool using a 1.5% gel and cleaned using the QiaQuick Gel Extraction protocol (Qiagen). Sequencing was performed by TrueSeq polyA stranded selection with 75 bp paired sequencing to extract 20 million reads. The samples analyzed included eight samples per sham and eight samples per IR injury (both sexes), with both scar and border zone samples of the myocardium (n = 16 total, n = 4 per zone per surgery).

#### 2.5.2. Gene Expression Data Analysis

RNA-seq data analysis was carried out by the CCHMC Division of Biomedical Informatics. The FASTQ files were obtained from the DNA Sequencing and Genotyping Core Facility at CCHMC. Quality control steps were performed to determine overall quality of the reads from the FASTQ files. Upon passing basic quality matrices, the reads were trimmed to remove adapters and low-quality reads using Trimmomatic [18].

The trimmed reads were mapped to the *Sus Scrofa* (swine) reference genome. Hisat2 was used for reference alignment. In the next step, transcript/gene abundance was determined using “Kallisto” [19]. A transcriptome index in Kallisto using Ensemble cDNA sequences for the pig was created. This index was then used to quantify transcript abundance in raw counts and transcripts per million (TPM). The quantified sample matrix was used for determining differential gene expression between experimental groups or to profile expression of a specific transcript across various conditions. The R package RUVSeq [20] was used to perform differential gene expression analysis between groups, with raw counts obtained from Kallisto used as input. Significant differentially expressed genes were obtained using a fold change cutoff of 2 and adjusted *p*-value/*p*-value cutoff of ≤0.05. The data files were uploaded to the GEO database (accession: GSE137293).

#### 2.5.3. Gene Ontology and Pathway Analysis

Downstream functional annotation of genes of interest and significantly dysregulated genes in the experiment were determined using gene ontology (GO) (cellular components, molecular function, and biological process) and pathway analysis. A detailed functional annotation and pathway analysis was performed using ToppFun tool from the ToppGene Suite [21]. An adjusted *p*-value cutoff of ≤0.05 was used to select functional annotations and pathways.

### 2.6. Statistical Analysis

All numerical data are presented as means ± SD, with *p* < 0.05 deemed significant. GraphPad Prism 8 software was used for statistical analysis, with Mann–Whitney, Kruskal–Wallis with Dunn’s post-hoc analysis, two-way ANOVA with Tukey post-hoc analysis, or Mixed-Effect Model (for repeated measures) with the Geisser–Greenhouse correction with Bonferroni post-hoc tests, performed as appropriate (as indicated in each Figure legend). All data were subject to Shapiro–Wilk normality testing prior to further analysis.

## 3. Results

### 3.1. Cardiac Ischemia/Reperfusion (IR) Injury, via Temporary Occlusion of the LAD, Produces Myocardial Tissue Damage in P30 Pigs

**Figure 1:**
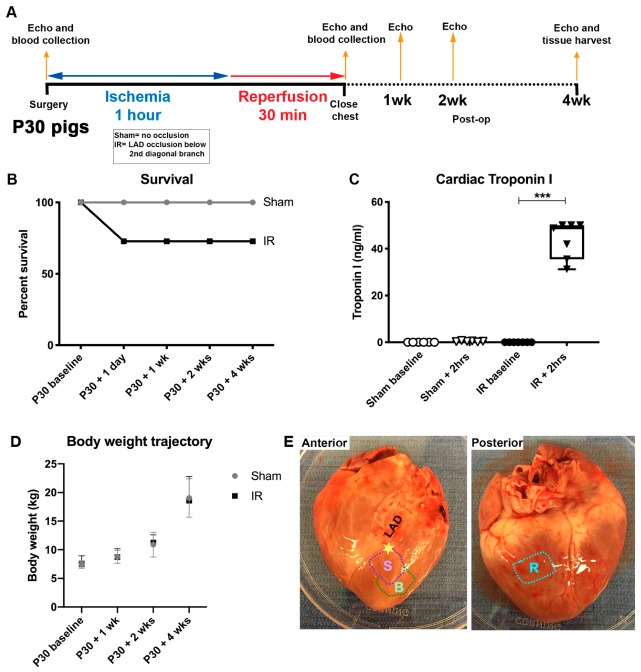
Cardiac ischemia/reperfusion injury assessment in P30 pigs. Schematic representation (**A**) of P30 pig ischemia/reperfusion (IR) procedure. Ischemia was induced for 1 h by occlusion of the left anterior descending artery (LAD) just distal to the second diagonal branch. Pigs were monitored for 30 min prior to closing the chest. Weekly/biweekly echocardiography (echo) assessments were undertaken up until tissue harvest at 4 weeks post-surgery. (**B**) Survival shown as a percent of the whole cohort in sham and IR pigs was assessed at regular intervals over the course of the study. (**C**) Cardiac Troponin I (ng/mL) was measured from blood plasma at baseline and 2 h following the initiation of ischemia or sham surgeries, to assess the extent of cardiac injury. (**D**) Body weights (kg) were measured prior to surgery, and then at regular time points post-surgery to assess growth trajectories of sham-operated and IR pigs over the 4 week study. (**E**) Image of a representative heart at 4 week tissue harvest, showing anticipated scar zone (S) and border zone (B) in relation to the occlusion site (represented as a star) just below the second diagonal branch of the LAD, and remote zone (R) taken from the posterior left ventricle. Data are mean ± SD. Two-way ANOVA with Tukey post-hoc analysis (**B**,**D**) and Kruskal–Wallis test with Dunn’s post-hoc analysis (**C**) were performed as appropriate, *** *p* < 0.001, n = 6–7/group Troponin I, n = 8/group body weight.

Since CM mitotic rates are similar at P0 and P30 in young swine [12], cardiac repair following temporary injury was assessed in P30 pigs. Blood collection and echocardiography were performed at baseline and subsequent time points over the course of the 4 week study (Figure 1A). On the day of the surgery, 3 out of 11 IR pigs did not survive (Figure 1B), due to cardiac arrest during the procedure or poor recovery following the cardiac injury. This rate of mortality was similar to previous reported models of IR injury [22]. Following day 1 of the study, all IR and sham pigs then survived to the end of the protocol.

Serum cTnI was increased >40-fold 2 h following ischemic injury in IR pigs compared with the sham-operated animals (Figure 1C), demonstrating an effective myocardial injury. Together these blood indicators, along with ST-segment changes on ECG, and observed myocardial color change, confirm myocardial tissue damage and ischemic injury in pigs subjected to the IR surgery.

No differences in body weight growth trajectories for the two groups were found over the course of the study protocol (Figure 1D). Thus, the ischemic cardiac injury, via temporary LAD occlusion, did not affect overall growth or development of the animals. At 4 weeks after surgery, the animals were sacrificed, hearts were excised, and left ventricular myocardium was isolated with scar, border, and remote zones collected as shown in Figure 1E. The scar zone was defined as immediately distal to the LAD suture site in both sham and IR pigs, and thus contained injured and viable myocardium. This IR protocol in P30 pigs produces a myocardial injury without significant morbidity or mortality of the operated animals.

### 3.2. Cardiac Function Declines Post-Ischemic Injury in P30 Pigs

**Figure 2:**
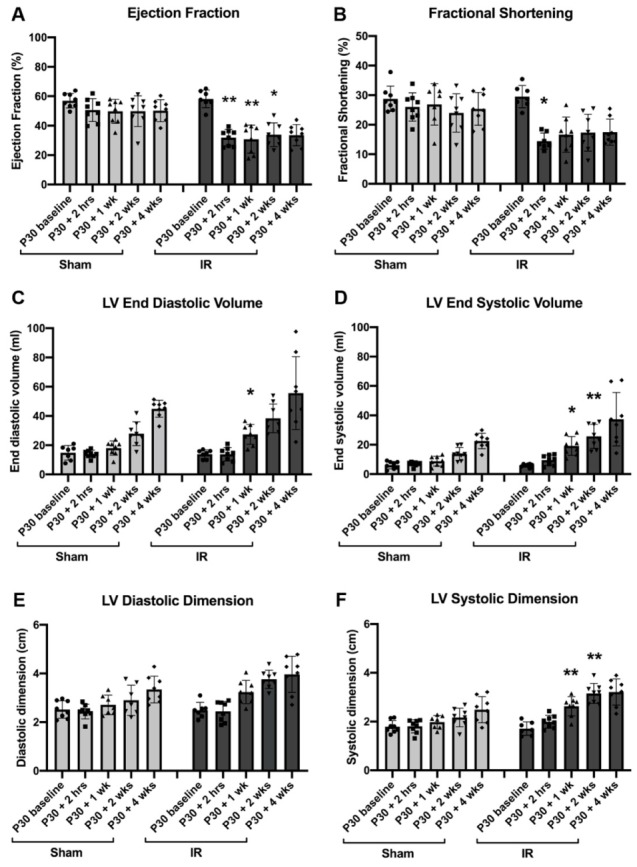
Cardiac function decreases at 2 h post-ischemia/reperfusion (IR) injury, but does not decline up to 2 months of age. Echocardiography analysis of sham-operated and IR pigs measured prior to surgery (P30 baseline), 2 h following the start of ischemia (or sham equivalent), 1 week, 2 weeks, and 4 weeks following surgery. (**A**) Ejection fraction (%), (**B**) fractional shortening (%), (**C**) left ventricular (LV) end diastolic volume (mL), (**D**) LV end systolic volume (mL), (**E**) LV diastolic dimension (cm) and (**F**) LV systolic dimension (cm). Data are mean ± SD. Mixed effect ANOVA analysis with Bonferroni’s post-hoc test * *p* < 0.05, ** *p* < 0.01 vs. same time point in sham, n = 8/group.

Echocardiographic assessment of cardiac function showed a decline in ejection fraction (EF, Figure 2A) and fractional shortening (FS, Figure 2B) in IR pigs from pre-operative baseline to 2 h post-injury. EF was similarly decreased at 1 and 2 weeks in IR compared to sham-operated animals. By the study endpoint, at 4 weeks post-surgery, EF and FS were unchanged compared with the assessment 2 h post-surgery for IR or sham-operated groups. Thus, the decrease in cardiac function was apparent by 2 h following IR, but did not improve or decrease further by 4 weeks post-injury.

Corresponding changes were observed in LV volumes and dimensions in systole and diastole, with increased size of the left ventricular chamber following injury (Figure 2C–F). However, ventricular septum and LV posterior wall thicknesses were unchanged between sham and IR pigs, suggesting a lack of muscular hypertrophic growth (Table 2). Assessment of LV size by echocardiography showed the expected growth increase as the pigs age. Stroke volume initially dropped post-injury, but was then similar between sham and IR pigs throughout the study, with heart rate being within the normal range (Table 2). These results indicate that, while cardiac function worsens immediately following IR in P30 pigs, a subsequent decline in heart function is not observed and heart growth parameters are in the normal range.

### 3.3. Cardiomyocyte Hypertrophy Is Not Induced with IR Injury, 4 Weeks Following Surgery

**Figure 3:**
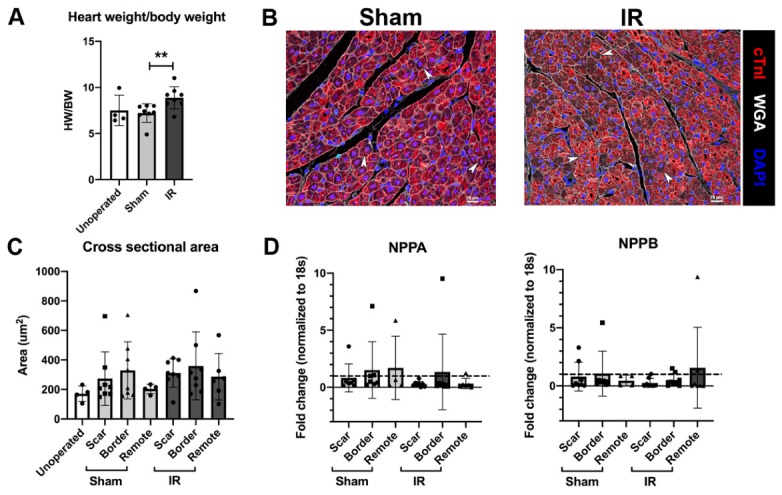
Cardiomyocytes (CMs) do not show hypertrophic growth 4 weeks after ischemia/reperfusion (IR) injury. (**A**) Heart weight corrected for body weight assessment in unoperated 2 month old controls, sham-operated and IR pigs at tissue harvest. (**B**) Representative myocardial scar region sections stained with cardiac Troponin I (cTnI, red), DAPI (blue) and wheat germ agglutinin (WGA, white), used for assessing cardiomyocyte cross sectional area (CSA), with arrowheads indicating examples of CM with central nuclei used for counting. (**C**) Quantification of CM CSA 4 weeks following surgery in unoperated controls, sham-operated and IR pigs divided into zonal regions: scar, border and remote. RT-qPCR for genes upregulated during cardiac hypertrophy (**D**) *NPPA* and *NPPB* mRNA in sham and IR pigs, with separated zones, normalized to *18S* and shown relative to unoperated 2 month control myocardial mRNA (dashed line). Data are mean ± SD. Mann–Whitney test (**A**) ** *p* < 0.01, 2-way ANOVA with Tukey post-hoc analysis (**C**,**D**; NS), n = 4–8/group. Scale bar = 20 µm.

Cardiac hypertrophy after IR injury was assessed by measurement of organ size and CM area. Heart weight/body weight (HW/BW), 4 weeks post-surgery, in IR pigs was increased compared with sham-operated or unoperated pigs (Figure 3A). However, histological assessment of CMs showed that cross sectional area of individual CMs was unaltered between unoperated control, sham and IR pigs, as well as amongst three different zones of the left ventricular myocardium (Figure 3B,C). Thus, CMs were of a similar size in all 2 month old pigs heart assessed, with and without ischemic injury. This is further confirmed with no differences between unoperated, sham and IR pig hearts in mRNA expression of *NPPA* and *NPPB* genes, that are upregulated during cardiac hypertrophy (Figure 3D). No evidence of hypertrophic growth following injury was also corroborated with no change in the posterior wall thickness of the LV between sham and IR pigs, via echocardiography assessment (Table 2). Our results therefore show that following ischemia, heart weight slightly increases relative to body weight, however CM hypertrophy is not observed as indicated by multiple parameters.

### 3.4. Cardiomyocyte Cell Cycling Activity Is Unchanged Following P30 Ischemia/Reperfusion Cardiac Injury

**Figure 4:**
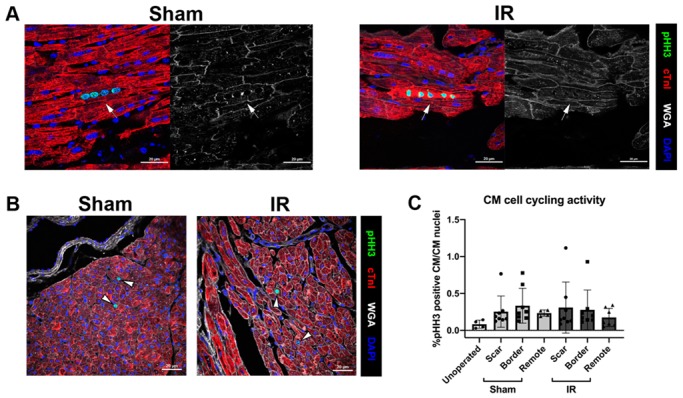
Cardiomyocytes (CMs) are multinucleated with cell cycling similar between sham and IR pigs at 4 weeks post cardiac surgery. (**A**) Immunohistochemistry of sham and IR scar regions of left ventricular myocardium at 2 months of age shown in the longitudinal orientation, highlighting multinucleation, as depicted by cardiac Troponin I (cTnI, red) in combination with DAPI (blue) with wheat germ agglutinin (WGA, white). In individual CMs with cell cycling activity, multiple nuclei show pHH3 immunoreactivity (green), indicated by the arrow. (**B**) Representative cross-section images of CM cell cycling in sham and IR (scar zones) pigs used for counting are shown with pHH3-positive CM nuclei indicated by white arrowheads. (**C**) Quantitation of %pHH3-positive CM, relative to total CM nuclei identified by cTnI+DAPI in unoperated control, sham, and IR pigs (three zones). Data are mean ± SD, analyzed by 2-way ANOVA with Tukey post-hoc analysis (C; NS). n = 4–8/group. Scale bar = 20 µm.

In unoperated pigs at 2 months of age, mitotic activity as identified by phospho-histone H3 (pHH3)-positive nuclei, is apparent in CMs as they become multinucleated [12]. Similar cell cycling in multinucleated CMs was observed in sham-operated or IR hearts, as measured by immunohistochemical staining of pHH3 with CMs identified by cardiac troponin I (Figure 4A). Interestingly, multinucleated CMs with each individual nucleus stained positive for pHH3 were found in sham, IR, and unoperated control cohorts (Figure 4A). To determine CM mitotic index, pHH3-positive CMs were assessed in cross-section, in which one pHH3 positive nucleus is apparent per CM (Figure 4B). No difference between sham and IR pigs was shown for CM cell cycling activity (Figure 4C), or between myocardial zones, suggesting that IR cardiac injury does not cause a change in CM proliferation. Thus, cell cycling is active in a subset of 2 month old pig CM, as indicated by pHH3 reactivity in CM nuclei, however cell cycle activity was unchanged between sham-operated and IR pigs.

It is important to note that these hearts were collected 4 weeks following surgery so any immediate effect of injury on CM proliferative capacity would not be detected if transient. However, assessment of cell cycling 4 weeks post-surgery suggests there is not an overall change in CM proliferative activity following ischemic cardiac injury.

### 3.5. Vessel Density and Cell Death Are Unchanged Following P30 Ischemia/Reperfusion Cardiac Injury

**Figure 5:**
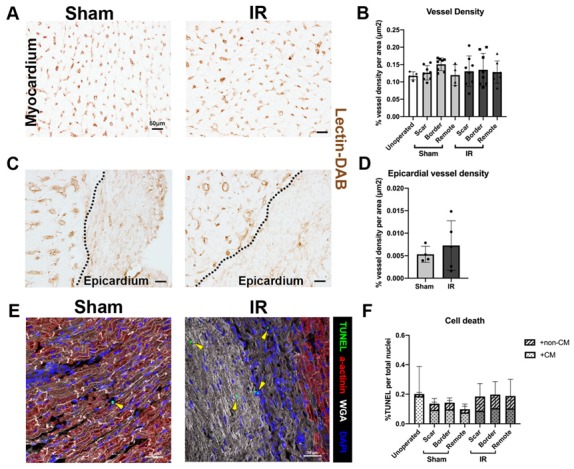
Vascular density and cell death are similar between sham and IR pigs at 4 weeks post-cardiac surgery. (**A**) Representative images of lectin-DAB stained microvessels in sham and IR myocardium (scar zone), with (**B**) quantification of unoperated control, sham, and IR (scar, border, and remote zones) pig vascular density, represented as % vessel density (lectin-DAB staining) per tissue area (μm^2^). (**C**) Epicardial regions of the hearts were stained for lectin-DAB to assess vascular changes within the region of most scar formation and (**D**) quantified in sham and IR pig scar zones. Dotted line represents edge of myocardium. (**E**) Representative images of cell death, as indicated by immunohistochemical TUNEL staining (green) with sarcomeric α-actinin (red) stained CM (yellow arrow heads, non-CM TUNEL positive cells), in sham and IR pigs (scar zones). (**F**) Quantitation of cell death in unoperated, sham, and IR (scar, border, and remote zones), expressed as a percentage of TUNEL positive cells per total nuclei (DAPI) and split based on the CM area (nuclei within cTnI positive region) and non-CM area (nuclei within cTnI negative region). Data are mean ± SD. Two-way ANOVA with Tukey post-hoc analysis (**B**,**F**) or Mann–Whitney tests (**D**) were performed as appropriate with no significant differences detected. n = 4–8/group. Scale bar = 50 μm (**A**,**C**), 20 μm (**E**).

Vascular density and cell death were assessed in the myocardium 4 weeks following injury as indicators of tissue damage and cardiac repair. Microvessel density, determined in lectin-DAB stained sections of the myocardium, was similar among unoperated control, sham-operated, and IR pigs at 2 months of age (Figure 5A,B). When the epicardial region was specifically assessed, there appeared to be a thickening of the epicardium with a lack of vascular density in both sham and IR pigs (Figure 5C). A high level of variability was seen within IR pigs in epicardial vascular density (Figure 5D), likely due to variable response to injury between individual pigs. Cell death, as assessed by TUNEL, was measured to determine whether or not cardiac injury had a damaging effect on cell survival. Cell death, 4 weeks following injury, was unchanged within the CM and non-CM region of the myocardium among unoperated control, sham-operated, and IR pigs (Figure 5E,F). Thus, IR in P30 pigs did not alter vascularity or myocardial cell death when assessed 4 weeks following injury.

### 3.6. The Immune Response Is Upregulated at 4 Weeks Following Cardiac Ischemic Injury

**Figure 6:**
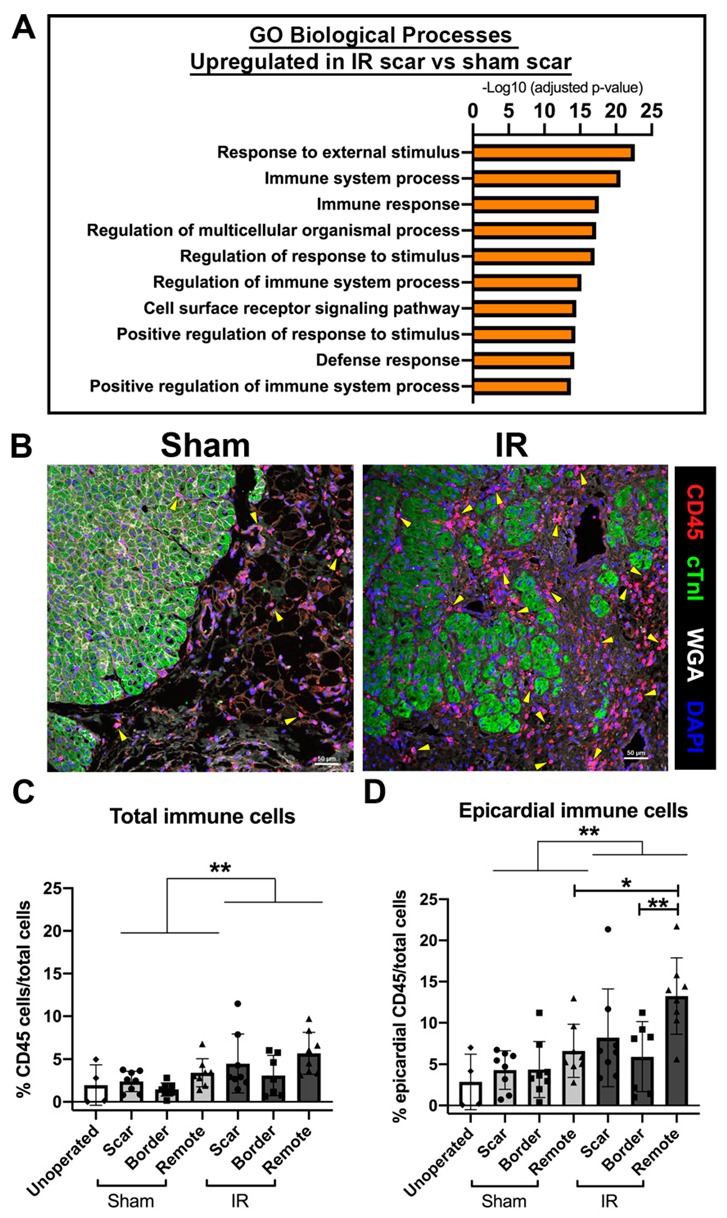
**The immune response is upregulated in the scar region of IR pigs 4 weeks following ischemic cardiac injury.** RNA-sequencing was undertaken on total RNA from sham and IR scar zones. (**A**) Unbiased gene ontology enrichments for biological processes that are upregulated in IR versus sham-operated pigs within the scar zone highlights an immune response to injury, n = 4 per group. (**B**) Representative images of sham-operated and IR scar regions (at the epicardial region with immunofluorescence for CD45-positive immune cells (red) alongside myocardium (cTnI, green), wheat germ agglutinin (WGA, white) and nuclei (DAPI, blue). Yellow arrowheads represent examples of CD45-positive immune cells. (**C**) Quantification of average myocardial immune cell expression, as indicated by CD45 immunohistochemistry (measured as a percentage of total cell nuclei) at 2 months of age across all zones of sham and IR pigs, as well as unoperated control myocardial tissue sections. (**D**) The epicardial region (as depicted in (**B**)) of each zone was also quantified (similar to (**C**)), as this was the area of most prominent scar formation proximal to the LAD ligation. Data are mean ± SD. Two-way ANOVA analysis (** *p* < 0.01) with Tukey post-hoc analysis, * *p* < 0.05, ** *p* < 0.01 n = 4–8/group. Scale bar = 50 μm.

To determine the differential gene expression changes between sham-operated and IR pigs, myocardial RNA was analyzed by RNA-sequencing (RNA-seq). Of particular interest were the gene expression changes within the expected scar region in sham-operated versus IR pigs. Unbiased GO term analysis revealed that biological processes typically related to an immune response were upregulated within the IR scar zone versus a comparable area in sham pig myocardium (Figure 6A). Such upregulated terms consist of immune system processing, response, and regulation (including *CD69*, *CD72*, *CD86*, *CD209*, *IL10*, *IL18*, *IL18R1*), as well as cell surface receptor signaling pathways and response to stimuli (including *CXCL9*, *CXCL10*, *CXCL11*, *CXCR3*). These main GO terms of differential gene expression associated with immune response were restricted to the ‘scar’ zone, as relatively fewer differences were found within the border zone of sham versus IR pigs. Thus, the majority of RNA transcriptional changes associated with inflammation in IR pigs occur close to the site of injury.

Immunohistochemical staining for CD45+ immune cells (all leucocytes) confirmed an upregulation of the immune response IR pigs compared with sham pigs, both within the myocardium and at the epicardial surface (Figure 6B–D). Following histological analysis 4 weeks following cardiac ischemic injury, increased numbers of CD45+ immune cells were found within the posterior remote region in IR pigs compared with the anterior of the heart close to the site of injury. Overall, RNA-seq and histological analysis shows increased immune response and presence of CD45+ cells 4 weeks following IR cardiac injury.

### 3.7. Myocardial Scarring Is Apparent 4 Weeks Following IR Cardiac Injury in P30 Pigs

**Figure 7:**
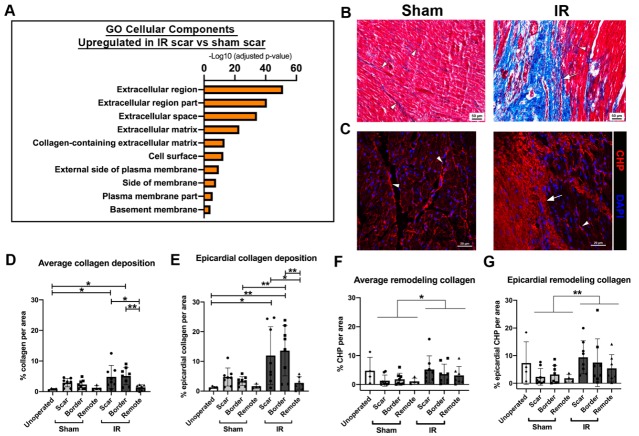
Scar formation is evident at 4 weeks post-surgery in ischemia/reperfusion (IR) pigs. RNA-sequencing was undertaken on total RNA from sham and IR scar zones. (**A**) Unbiased gene ontology enrichments for cellular components that are upregulated in IR versus sham-operated pigs, within the scar zone, showing ECM gene expression changes in response to injury. (**B**) Representative Masson’s trichrome staining (myocardium = pink, collagen = blue), in sham-operated and IR scar region sections. The arrow depicts obvious scar formation in IR pigs, with arrowheads showing interstitial collagen in sham-operated and IR myocardial sections. (**C**) Representative images in sham and IR scar zone of immunohistochemistry for collagen hybridizing peptide (CHP, Helix3), marking remodeling collagen (red) alongside DAPI for nuclei (blue). The arrow depicts dense scar area, with arrowheads indicating interstitial remodeling collagen (red). (**D**) Quantification of average collagen deposition as indicated by Masson’s trichrome staining at 2 months of age across all zones of sham and IR pigs, as well as unoperated control myocardial tissue sections. Collagen (blue staining) was measured relative to tissue area (μm^2^) and displayed as a percentage. (**E**) The epicardial region of each zone was also quantified (similar to (**D**)), as this was the area of most prominent scar formation proximal to the LAD ligation. (**F**) Quantification of average CHP deposition at 2 months of age across all 3 zones of sham and IR pigs, as well as unoperated control myocardial tissue sections. CHP (red) was measured relative to tissue area (μm^2^) and displayed as a percentage. (**G**) The area of CHP reactivity per total area in the epicardial region of each zone was also quantified. Data are mean ± SD. Two-way ANOVA analysis with Tukey post-hoc analysis, * *p* < 0.05, ** *p* < 0.01 n = 4–8/group. Scale bar = 50 μm (**B**), 20 μm (**C**).

To determine whether cardiac ischemic injury at P30 resulted in scar formation, myocardial samples were assessed for gene expression levels of ECM related genes as well as histological staining. From RNA-seq, unbiased GO term analysis revealed that the cellular components associated with ECM regulation were upregulated in IR scar versus sham scar zone tissue (Figure 7A).

Scar formation and collagen remodeling were assessed after IR injury. Histological analysis of fibrillar collagen by Masson’s trichrome staining scar zones in all pigs demonstrated an obvious scar at the epicardial region in IR pigs, that was not observed in sham-operated animals (Figure 7B). When multiple images per zone of the myocardium were stained for collagen deposition, collagen accumulation was increased by >5-fold in the scar and border zone of IR pig hearts, compared to unoperated 2 month old control pigs (Figure 7D). As expected, the remote zone of IR pigs showed less collagen deposition than the scar and border zones. The most prominent scarring was obvious at the epicardial region of the myocardium, thus, when this area was quantified, there was an increase in collagen deposition in IR pigs versus sham (Figure 7E). As expected, the remote zone had less collagen deposition than the epicardial region, consistent with greater myocardial injury in the anterior region of the heart proximal to the LAD occlusion. Although not significant, trending increases in collagen deposition in sham-operated animals (Figure 7D,E) may be indicative of mild cardiac injury during the process of opening the chest cavity and placing a suture in the myocardium (without occlusion). However, large regions of collagen deposition at the site of injury were only observed in animals subjected to IR.

Immunohistochemical staining for collagen hybridizing peptide (Helix3), for detection of remodeling collagen, illustrated a similar obvious scar formation in IR pigs versus sham (Figure 7C,F). Again, the greatest change in ECM response between sham and IR pigs, as indicated by remodeling collagen, was apparent in the epicardial region of the myocardium (Figure 7G). 2 month old unoperated control pigs showed a variable level of remodeling collagen, hence no significant differences between this cohort and the surgical groups were observed. Taken together, IR injury via LAD occlusion in P30 pigs creates obvious scar generation with ECM remodeling at 4 weeks post-IR. This is most apparent at the area closest to the occlusion site predominant on the epicardial surface.

## 4. Discussion

While there is extensive information available on postnatal heart development and regenerative potential after injury in rodents [2,8,9,10,11], much less is known about cardiac repair and regeneration in large mammals, including humans [1]. Here, we report cardiac repair related to scar formation following transient ischemia/reperfusion injury in P30 pigs. We demonstrate that P30 pigs lack the capacity of cardiac regeneration through production of new CMs following IR, despite detectable mitotic activity in a subset of CMs at this stage [12]. Myocardial damage is evident immediately following IR injury, with a decrease in cardiac function detected 2 h after injury, that does not improve or worsen over the 4 week study period. While cell cycling in multinucleated CMs is evident in unoperated, sham-operated, and IR pigs at 2 months of age, CM cell cycle activity is not increased 1 month post-injury. Cardiac repair is evident in the pigs subjected to IR with increased inflammation and the formation of a localized scar in the epicardial region proximal to the region of ischemic injury. This study therefore demonstrates that transient ischemic injury in the hearts of young swine leads to decreased cardiac function and increased scarring, but not increased proliferation of CMs.

The temporary occlusion of the LAD, immediately distal to the second diagonal branch, results in a significant decrease in cardiac function, as measured by ejection fraction and fractional shortening 2 h after surgery. Such a decrease in function is similar to previously reported swine models of LAD ligation and permanent myocardial infarction (MI) [3,4,23,24,25,26,27,28]. The decreased cardiac function after transient IR was then maintained to 4 weeks post-injury, with no obvious morbidity or mortality beyond the day of the surgery. In an adult pig model of IR, ejection fraction is maintained at a comparable decreased level from baseline to 2 months post-surgery [26]. By 3 months post-IR there is a further decrease in cardiac function, alongside CM hypertrophy, suggesting heart failure progression in adult pigs. In contrast, IR injury in P30 pigs did not lead to changes in chamber wall morphology or CM cross sectional area, suggesting a lack of pathologic cardiac hypertrophy 4 weeks following surgery. This transient cardiac injury model therefore is effective in showing that even a mild injury (1 h of ischemia) can lead to decreased cardiac function, which does not worsen over time, in addition to a cardiac healing response apparent in inflammation and scarring.

When assessed at 2 months of age, sham-operated and IR pigs showed evidence of mitotic activity (pHH3 expression) and ongoing multinucleation in approximately 0.35% of CMs. This confirms findings from our lab that show low, but detectable, CM mitotic activity in a subset of CMs at 2 months of age [12]. In mice, CMs undergo binucleation, cell cycle arrest, and lose their capacity to regenerate approximately 1 week after birth [2,7,29]. A previous study of neonatal pigs demonstrated a similar loss of regenerative response after P3 following a more severe cardiac injury by permanent MI [3,4]. Here we show that a milder transient cardiac ischemic injury at P30 also does not lead to increased CM cell cycling 4 weeks following surgery, even though a low percentage of mitotic CM nuclei were detected at the time of injury. Taken together, the CM mitotic activity in young swine during multinucleation is not sufficient to produce cardiac regeneration beyond a few days after birth, similar to what has been reported for rodents.

The cardiac injury response in young swine after the postnatal period includes inflammation and scarring. Young pigs after cardiac injury produce an immune response to initiate cardiac healing and subsequent ECM remodeling, as has been observed in older large and small mammals [3,4,30,31]. With transient ischemic injury in P30 pigs, we observed increased inflammation throughout the heart with localized ECM upregulation and thickening of the epicardial collagen matrix at the site of injury. Similar epicardial activation and thickening has been observed in adult mice and humans after cardiac ischemic injury that may be part of the mammalian cardiac injury response [32,33]. Here we show that a mild transient ischemic injury at P30 in pigs also leads to localized scarring at the site of injury. Of note, the process of opening the chest and placing a suture (with no occlusion), as in sham pigs, creates a degree of surgery-associated injury response similar to IR pigs. In contrast to permanent MI after P3, the scar generated in pigs with transient IR at P30 did not span the entire myocardium, however, the milder injury resulted in a small regional scar in hearts with reduced EF observed within 2 h post-IR. The ischemic damage to the myocardium does not recover after injury and is likely to be the major contributor to prolonged reduced cardiac output. In addition, scar generation and ECM remodeling apparent after cardiac injury may be inhibiting any reparative/regenerative response to cardiac injury beyond the postnatal period in mammals.

Together with previous reports utilizing a permanent cardiac injury model [3,4], here our transient ischemic model demonstrates inflammation and scarring, not CM renewal in young pigs, despite presence of mitotic activity in a subset of CM. Any potential of cardiac regeneration in young large mammals may be hindered by scar development in the injured heart, rather than cell cycle withdrawal and binucleation as previously suggested [3,34]. Loss of cardiac regenerative capacity has been linked to oxidative stress at birth, ECM maturation, and infiltration of macrophages in addition to CM cell cycle exit [2,3,4,35,36]. Further understanding the factors which inhibit regeneration in the developing pig will add to the current limited knowledge on mechanistic regulation of cardiac repair following injury in young large mammals. This improved knowledge, through use of models as described in this study, may aid in identifying mechanisms to test reactivation and promotion of heart regeneration in adults. However, translation of pig models to humans in regards to identifying cardiac therapeutics focused purely on CM proliferative reactivation must proceed with caution. Differences in the cell biology of CM between species will likely limit the use of pigs in pre-clinical studies.

## Figures and Tables

**Table 1:** Porcine primer sequences used for RT-qPCR analysis of pig myocardial mRNA normalized to *18S* ribosomal RNA.

RNA	Sequence 5′-3′	
	Forward	Reverse
*18S*	AATTCCGATAACGAACGAGACT	GGACATCTAAGGGCATCACAG
*NPPA*	TGAACCCAGCCCAGAGAGAT	CAGTCCACTCTGTGCTCCAA
*NPPB*	GTTGCTGCTAGGATGCCGTT	TACCTCCTGAGCACATTGCAGC

**Table 2:** Tissue doppler echocardiography measurements of P30 pigs subjected to sham or ischemia/reperfusion (IR) surgery, measured at baseline (prior to surgery), 2 h, 1 week, 2 weeks, and 4 weeks after surgery in 2D four-chamber and M-mode images.

Parameter	Surgery	P30 Baseline	P30 + 2 h	P30 + 1 Week	P30 + 2 Weeks	P30 + 4 Weeks
Ventricular Septum Diastolic Thickness (cm)	Sham #	0.43 ± 0.04	0.41 ± 0.09	0.51 ± 0.12	0.59 ± 0.07	0.59 ± 0.12
	IR	0.48 ± 0.10	0.43 ± 0.06	0.47 ± 0.20	0.55 ± 0.07	0.58 ± 0.14
Ventricular Septum Systolic Thickness (cm)	Sham	0.70 ± 0.15	0.61 ± 0.15	0.76 ± 0.10	0.93 ± 0.14	0.90 ± 0.27
	IR	0.79 ± 0.14	0.53 ± 0.06	0.72 ± 0.24	0.70 ± 0.15	0.76 ± 0.26
LV Posterior Wall Diastolic Thickness (cm)	Sham	0.44 ± 0.06	0.47 ± 0.03	0.46 ± 0.08	0.56 ± 0.09	0.56 ± 0.09
	IR	0.42 ± 0.04	0.44 ± 0.06	0.47 ± 0.11	0.55 ± 0.14	0.55 ± 0.17
LV Posterior Wall Systolic Thickness (cm)	Sham	0.62 ± 0.10	0.66 ± 0.07	0.74 ± 0.08	0.80 ± 0.10	0.88 ± 0.13
	IR	0.67 ± 0.10	0.68 ± 0.11	0.75 ± 0.18	0.81 ± 0.21	0.88 ± 0.20
Stroke Volume (mL)	Sham	8.27 ± 2.86	7.14 ± 1.70	9.21 ± 2.44	14.09 ± 5.70	22.35 ± 3.38
	IR	8.16 ± 2.23	4.27 ± 1.38 **	8.23 ± 2.69	12.66 ± 3.14	18.24 ± 8.09
Heart Rate (bpm)	Sham	145.28 ± 20.46	107.22 ± 10.05	177.50 ± 20.84	147.44 ± 27.85	131.11 ± 10.59
	IR	149.72 ± 28.44	126.52 ± 34.15	183.71 ± 27.90	171.14 ± 13.37 *	147.62 ± 18.11

**#** Data are shown as mean ± SD, with mixed-effect ANOVA model with post-hoc Bonferroni tests * *p* < 0.05, ** *p* < 0.01 vs sham surgery at equivalent time. All parameters shown are significantly different over time as the pigs age (via mixed effect model analysis for repeated measures, thickness parameters *p* < 0.01, stroke volume and heart rate *p* < 0.0001). n = 4–8/group.
